# Perspectives from cystinosis: access to healthcare may be a confounding factor for variant classification

**DOI:** 10.3389/fgene.2024.1402667

**Published:** 2024-07-24

**Authors:** Chen-Han Wilfred Wu, Alicja Tomaszewski, Louisa Stark, Fernando Scaglia, Ewa Elenberg, Fredrick R. Schumaker

**Affiliations:** ^1^ Department of Genetics and Genome Sciences, Case Western Reserve University School of Medicine and University Hospitals, Cleveland, OH, United States; ^2^ Department of Urology, Case Western Reserve University School of Medicine and University Hospitals, Cleveland, OH, United States; ^3^ Department of Human Genetics, University of Utah School of Medicine, Salt Lake City, UT, United States; ^4^ Department of Molecular and Human Genetics, Baylor College of Medicine, Houston, TX, United States; ^5^ Department of Pediatrics, Baylor College of Medicine, Houston, TX, United States; ^6^ Department of Population and Quantitative Health Sciences, Case Western Reserve University School of Medicine, Cleveland, OH, United States

**Keywords:** cystinosis, population genetics, healthcare disparities, kidney stone, health equity, pathogenicity

## Abstract

Genetic variability persists across diverse populations, and it may impact the characterization of heritable diseases in different ancestral groups. Cystinosis is a metabolic disease caused by pathogenic variants in the *CTNS* gene causing the cellular accumulation of cystine. We attempted to assess the currently poorly characterized prevalence of cystinosis by employing a population genetics methodology. However, we encountered a significant challenge due to genetic variations across different populations, and the consideration of potential disparities in access to healthcare made our results inconclusive. Pathogenic *CTNS* variants were identified in a representative global population cohort using The Human Gene Mutation Database (HGMD) and the 1000 Genomes (1 KG) database. The c.124G>A (p.Val42Ile) variant was reported to be pathogenic based on an observation in the white population presenting with atypical phenotypes, but it would be reclassified as benign in the African ancestral group if applying the ACMG allele frequency guideline due to its high allele frequency specifically in this population. Inclusion or exclusion of this c.124G>A (p.Val42Ile) variant results in a significant change in estimated disease prevalence, which can impact the diagnosis and treatment of affected patients with a broad range of phenotypic presentations. This observation led us to postulate that pathogenic manifestations of the disease may be underdiagnosed due to variable expressivity and systemic inequities in access to care, specifically in the African subpopulation. We call for a more cautious and inclusive approach to achieve more equitable care across diverse populations.

## 1 Introduction

Cystinosis is a rare autosomal recessive disorder that is characterized by systemic accumulation of cystine crystals in different types of cells and tissues. It is the most common inherited cause of Fanconi syndrome observed in children (Elmonem et al., 2016a). In cystinosis, cystine crystals begin to accumulate in the lysosomes due to impaired transport out of lysosomes.

The incidence of cystinosis varies, ranging from 1 in 115,000 live births to 1 in 260,000 live births (Ebbesen et al., 1976; Hult et al., 2014). Several populations have been identified that suggest an increased incidence rate due to founder mutations or in communities in which consanguinity is more common (Elmonem et al., 2016b). The prevalence of cystinosis is poorly characterized, and the population-specific data is missing. The best estimate is based on the fact that there are an estimated 500–600 patients in the United States diagnosed with cystinosis, corresponding to a prevalence of 1 in 479,000 to 1 in 575,000 (Gahl et al., 2002; Doyle and Werner-Lin, 2015).

From a genetics perspective, the presentations of cystinosis correlated with the categories of disease-causing variants can be grouped into 4 classifications (Kalatzis et al., 2004): infantile cystinosis, juvenile cystinosis, ocular cystinosis, and atypical cystinosis. Infantile cystinosis is most common and correlated with severe disease-causing variants in which symptoms manifest at a young age and typically involve end stage renal disease without treatment. Juvenile cystinosis affects fewer patients and presents with mild renal symptoms compared to the infantile form, but it can still cause permanent renal damage and possible end stage renal disease. The ocular form rarely presents until adulthood, and it primarily involves symptoms including photophobia related to the accumulation of cystine but may also cause milder renal damage. Atypical cystinosis captures the range of severity of presentations between the infantile and juvenile forms.

In nephropathic cystinosis, the most severe form of cystinosis, symptoms appear several months after birth (Gahl, 1986; Gahl et al., 2002). Patients present with failure to thrive, polyuria, and rickets. Laboratory work up shows similar characteristics to Fanconi syndrome with hyperchloremic metabolic acidosis and urinary loss of low molecular weight protein, glucose, amino acids, phosphate, calcium, sodium, potassium, bicarbonate, carnitine, and water (Gahl et al., 2002). Patients may present with a milder renal manifestation that consists of nephrotic range proteinuria with no Fanconi syndrome, similarly to Dent disease with multinucleated podocytes visible in the kidney biopsy (Chandra et al., 2010).

The etiology of cystinosis has been identified as a monogenic disease from biallelic pathogenic variants in the *CTNS* gene located on chromosome 17p13 (McDowell et al., 1995). *CTNS* encodes the carrier membrane protein cystinosin, which aids in the transport of cystine out of the lysosomal compartment (Sumayao et al., 2018). Deficiency or defect in cystinosin causes cystine to build up in the lysosomes of tissues resulting in crystal formation (Sumayao et al., 2018). Cystine crystal accumulation occurs throughout the lifetime in most cells and tissues of the body including the conjunctiva, corneas, liver, spleen, lymph nodes, kidneys, thyroid, intestines, rectal mucosa, muscle, brain, macrophages, and bone marrow (Gahl et al., 2002). Earlier manifestations of disease include growth restriction, polyuria, and feeding intolerance, and progress to nephrocalcinosis, kidney failure, and photophobia if untreated. The kidney is the first organ affected in nephropathic cystinosis, making early diagnosis and treatment critical in preserving renal function.

Diagnosis of cystinosis relies on measuring leukocyte cystine content or identifying corneal crystals on slit-lamp examination (Nesterova and Gahl, 2013). However, crystals may not form until 1 year after birth (Gahl et al., 2000). Cystinosis can also be confirmed by genetic assays identifying variants in the *CTNS* gene. Studies have shown that diagnosing cystinosis at an early age by integrating it into newborn screening programs followed by appropriate treatment is critical to limit the systemic damage caused by the disease (Hohenfellner et al., 2022). However, access to diagnostic testing is significantly reduced in low-income countries. In 2022, access to genetic analysis was 63% and leukocyte-cystine level measurement was 30% in countries with developing economies, compared to 100% and 95% in developed countries, respectively (Regnier et al., 2023).

The most common treatment for cystinosis is the use of cysteamine to decrease levels of cysteine accumulation to delay disease progression and the development of extra-renal pathologies (Gahl et al., 1987). However, it is not effective as a cure for the presentation of Fanconi syndrome in these patients (Ivanova et al., 2014). In the past without effective available therapies, patients would die of renal failure by 10 years of age. With therapeutic interventions that have been available for the past 5 decades, the lifespan for renal function has doubled (Gahl et al., 2002). Eventually, all nephropathic cystinosis patients will require renal replacement therapy, combined with continued treatment as cystine crystals continue to deposit in different organs (Nießl et al., 2022). Although the lifespan of affected patients has improved significantly with therapeutic interventions, progressive complications develop from different organ involvement (Ariceta et al., 2019). Importantly, access to these treatment options remains variable globally especially in low-income countries. In 2022, cysteamine eyedrops had a 63% availability and cystine-reducing medication (Procysbi) had a 7% availability in low-income countries compared to 95% and 74% availability, respectively, in developed countries (Regnier et al., 2023).

In order to evaluate the feasibility of newborn screening for cystinosis to propose an informed public health policy, our initial aim was to better understand the genetic prevalence of cystinosis by investigating pathogenic variants in the population database. We identified specific pathogenic variants that are carried by the healthy population that may facilitate earlier therapeutic intervention. However, we discovered differences in variant allele frequencies across populations in various ancestral groups which raised questions on the classification of pathogenicity of some variants based on their allele frequency. This finding supports our proposal that access to healthcare in different populations may affect the classification of allele pathogenicity, especially with alleles that have incomplete penetrance and variable expressivity. This study supports our suggestion to increase efforts in the diagnosis of cystinosis in diverse populations, especially among those who are affected by systemic decreased access to healthcare, to better characterize the natural history of pathogenic alleles with different frequencies among various populations towards a goal of more equitable care.

## 2 Methods

Characterizing the prevalence of cystinosis as a rare autosomal recessive disease through cohort studies is challenging, limited by the number of affected individuals in the population. However, utilizing the 1000 Genomes database allows us to compute the carrier rates and allele frequencies of *CTNS* pathogenic variants in the healthy population. We can estimate the affected rates using the principles of Hardy-Weinberg equilibrium and the relevant carrier rate and allele frequency (Hardy, 1908). This methodology has been validated in previous studies (Schrodi et al., 2015; Bainbridge, 2020) and has been applied in estimating the prevalence of RAG deficiency (Chen et al., 2014) and primary hyperoxaluria (Hopp et al., 2015).

*CTNS* variants in the 1000 Genomes (1 KG) database were identified using the same methods as outlined in our previous study (Wu et al., 2023). The 1 KG database is a comprehensive database of genomes from healthy individuals in multiple representative populations around the world, identifying 98% of alleles with a frequency of more than 1% in these populations, in addition to some lower frequency alleles (1000 Genomes Project Consortium et al., 2012). Phased VCFs aligned to the human reference genome from the 1000 Genomes Project were downloaded from https://www.internationalgenome.org/data-portal/. Sample IDs corresponding to the 1092 unrelated individuals from the 1000 Genomes Phase I dataset (1000 Genomes Project Consortium et al., 2015) were determined from that release’s panel file (https://ftp.1000genomes.ebi.ac.uk/vol1/ftp/phase1/analysis_results/integrated_call_sets/integrated_call_samples.20101123.ALL.panel). This includes 4 main superpopulations: African, Admixed American, East Asian, and European. There are 14 subpopulations within these superpopulations including: Colombian in Medellin, Colombia; Iberian populations in Spain; Toscani in Italy; Mexican ancestry in Los Angeles, California; British in England and Scotland; Yoruba in Ibadan, Nigeria; Japanese in Tokyo, Japan; Utah residents (CEPH) with Northern and Western European ancestry; Han Chinese in Beijing, China; and Luhya in Webuye, Kenya. The genomic coordinates of *CTNS* were used to subset the corresponding chromosome VCF file. This work was completed with the High Performance Computing Resource in the Core Facility for Advanced Research Computing at Case Western Reserve University.

Known *CTNS* pathogenic variants associated with cystinosis-related symptoms were obtained from the Human Gene Mutation Database (HGMD) ((HGMD^®^ Professional, http://www.hgmd.cf.ac.uk/ac/index.php). VCF files were exported after limiting the genes to *CTNS*.

Variants present in both databases were compared and intersected to identify disease-causing variants and their carriers in the general population. Allele frequencies were calculated from the 1 KG database, and the expected carrier rate and affected rate were estimated using Hardy-Weinberg equilibrium.

We further manually reviewed pathogenic variants identified in the 1 KG population based on ACMG guidelines based on allele frequency of each variant in ancestral group populations. Allele frequencies from various ancestral groups were sourced from gnomAD v4.0.0 (Karczewski et al., 2020). Reclassification of pathogenicity is presented if there are conflicts between HGMD and ACMG.

## 3 Results and dilemma

*CTNS* variants were procured from the HGMD and 1 KG databases. Using the 1 KG database, 444 unique CTNS variants were found among 1092 healthy individuals. The HGMD database was used to identify 75 variants known to be associated with a pathology of cystinosis. The HGMD and 1 KG databases were intersected to identify 2 disease-causing variants that were present in the general population (Table 1). A total of 36 individuals carried these 2 disease-causing variants (Table 1).

**TABLE 1:** Characteristics of intersected genetic variants (pathogenic variants) of *CTNS* in 1 KG and HGMD, including allele frequency in specified ancestral groups.

Chromosome	Position	Variant Identifier	Reference allele	Variant allele	C. Nomenclature	P.Nomenclature	Exon number	Type	Number of people carrying this variant (all heterozygous)	Observed allele frequency (%)
chr17	3550800	rs35086888	G	A	c.124G>A	p.Val42Ile	4/13	missense	35	35/2184 (1.60%)
chr17	3559792	rs113994206	T	C	c.473T>C	p.Leu158Pro	8/13	missense	1	1/2184 (0.04%)

Ancestry group	Allele frequency
African	0.06261
European (non-Finnish)	0.00006186
European (Finnish)	0
East Asian	0.00002228

Chromosome position based on Genome Reference Consortium Human Build 37 (GRCh37, hg19).

1 KG: 1000 Genomes Project Phase 3; HGMD: Human Gene Mutation Database SNV: single nucleotide variant.

The first variant, c.124G>A, has been found to cause atypical cystinosis, which has incomplete penetrance and variable expressivity; phenotypic presentation among individuals with this allele can vary in severity. The c.473T>C variant has been found to cause infantile nephropathic cystinosis that presents with the severe cystinosis phenotype in infancy. Among the 36 carriers, 35 individuals were shown to carry the c.124G>A variant, and the c.473T>C variant was carried by 1 individual in the 1 KG database (Table 1). Upon further investigation of this variant in gnomAD v4.1.0, we found that the allele frequency of the variant of interest was 6.26% in the African population. We compared this to the allele frequencies of 0.006% in the European (non-Finnish) population, 0 in the European (Finnish) population, and 0.002% in the Asian population for the same variant to underscore the notable difference in specifically the African population.

According to ACMG guidelines, an allele frequency of more than 5% is considered to be stand-alone support (BA1). The allele frequency of the c.124G>A variant in the African ancestral group is 6.26%, and it is therefore classified as benign. However, we present this finding as a potential dilemma as the allele frequency of this variant is only increased in the African ancestral group (6.26%); in all other ancestral groups in 1 KG, its allele frequency does not classify it as benign.

**FIGURE 1:**
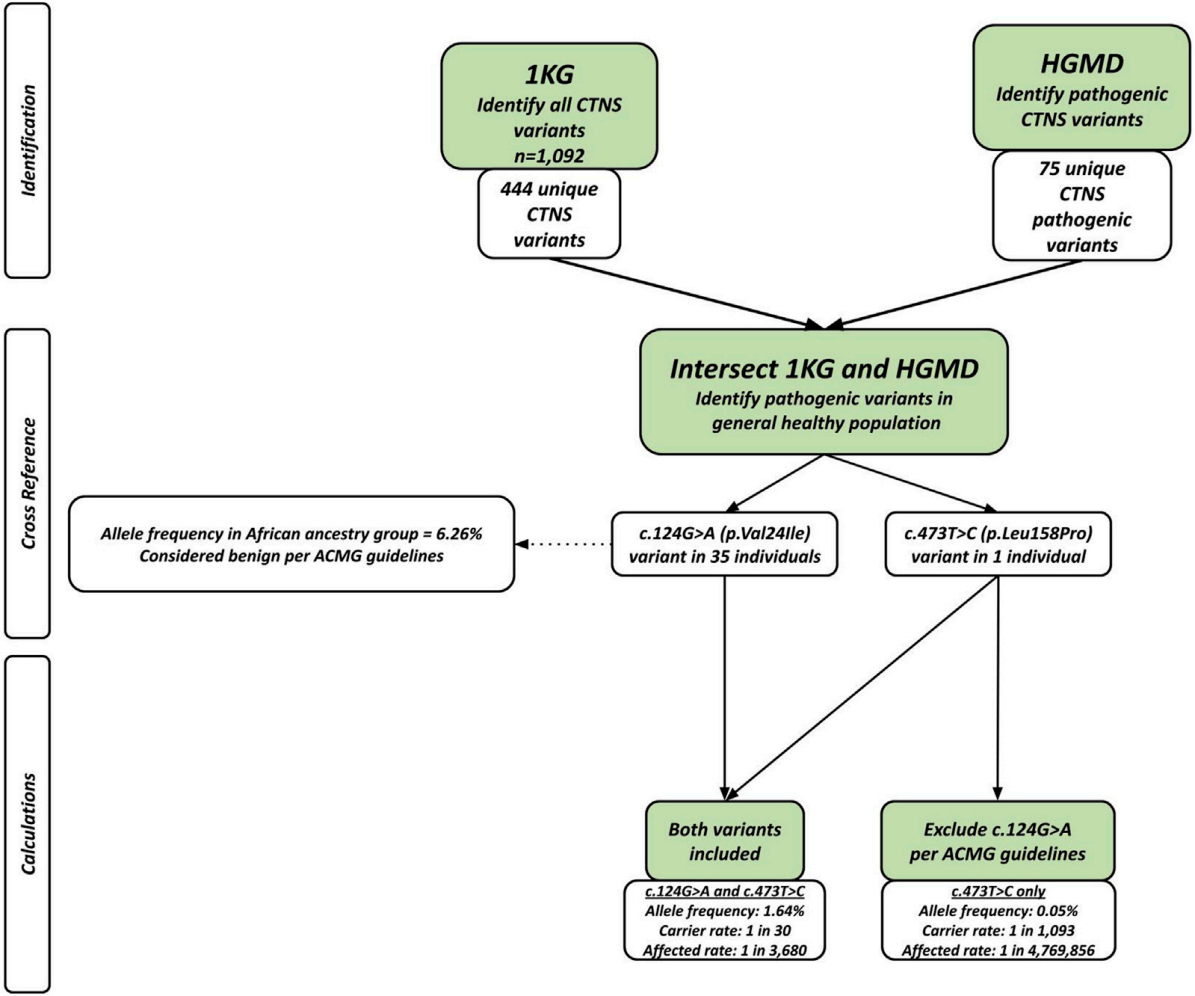
Flowchart showing populations genetics method applied in study. After intersecting the two databases, there were 36 people sharing 2 different pathogenic variants. The allele frequency and carrier rate calculations differed based on whether the c.124G>A (p.Leu158Pro) was included or excluded.

Based on these results and supported by the established evidence of healthcare disparities that disproportionately negatively impact the African population in addition to the poor characterization of cystinosis prevalence and missing information in the African population, we present the concern of whether to classify the c.124G>A variant as benign or pathogenic. With this dilemma, two separate calculations were performed using the Hardy Weinberg Equilibrium for allele frequency and carrier and affected rates: one including both variants and another excluding the c.124G>A variant per ACMG guidelines (Figure 1). With both variants included, the allele frequency was calculated as 1.64%, and the carrier and affected rates were calculated as 1 in 30 and 1 in 3,680, respectively. Excluding the c.124G>A variant, the allele frequency was calculated as 0.05%, and the carrier and affected rates were calculated as 1 in 1,093 and 1 in 4,769,856, respectively.

## 4 Discussion

The purpose of this study is to present the dilemma that the variability in allele frequency between different ancestral groups and how variants are categorized as benign or pathogenic based on previous reports *versus* current guidelines, and call for a more cautious and inclusive approach, specifically considering the c.124G>A variant in *CTNS* causing cystinosis. A PheWAS or further phenotypic analyses would help to ascertain the true nature of a variant like this.

The c.124G>A variant of interest included in this study is one of the earliest pathogenic variants identified in 1999 (Attard et al., 1999) and it was shown to be associated with atypical cystinosis associated with corneal dysfunction only (Attard et al., 1999). However, according to ACMG guidelines, there is supporting evidence suggesting that the c.124G>A variant should be excluded as a pathogenic variant due to its high allele frequency in the African population (Harrison et al., 2019). However, it is important to note that this increased allele frequency is only present in the African subpopulation in the 1 KG database and absent in other subpopulations (Figure 1).

This variant was known to cause atypical cystinosis, known to show incomplete penetrance and variable expressivity which can manifest as a broad range of phenotypic presentations in carrier individuals (Attard et al., 1999). Although the patient may not initially present with symptoms, they may still become symptomatic later, and family members who also inherit the variant may present with different phenotypic severity as well. In addition to HGMD, OMIM, a manually curated database of pathogenic variants of genes that is regularly updated, categorizes the c.124G>A as pathogenic and causative of atypical cystinosis. Identifying these patients especially in a higher risk population may benefit from early follow up and intervention if symptoms arise.

The fact that the c.124G>A variant is only present at a high allele frequency in the African population raises the question of health equity and racial disparities in care. Bias and decreased access to equitable care among non-white populations has been shown across various medical specialties (Williams and Rucker, 2000). If these individuals are at greater risk of receiving less equitable care due to bias or decreased resources, it is possible that the diagnosis and identification of symptoms are being missed, especially if they present in the less severe phenotype of atypical cystinosis. If individuals with the c.124G>A variant that is represented at an increased allele frequency in the African population do not receive adequate care to identify cystinosis-associated symptoms causing them to be labeled as asymptomatic, the variant would be inappropriately classified as benign.

There may also be a discrepancy in the representation of ethnic groups in genetic studies. Studies suggest that there is greater hesitancy among some ethnic minorities to utilize genetic analyses, which decreases the availability of clinically useful data for these groups (Saulsberry and Terry, 2013). Notably, there is a significantly greater genetic and linguistic diversity among the African population compared to populations from other continents, which is not accurately represented by the low proportion of participants of African ancestry in the GWAS Catalog (Fatumo et al., 2022). There is an existing consensus in the literature that we should sequence more non-white populations (Buniello et al., 2019). It has been well-established in literature that large geographic regions including those in Sub-Saharan Africa are significantly underrepresented in genetic studies of disease including cystinosis (David et al., 2019). There have been multiple variants identified in these populations linked to the development of cystinosis-related symptoms, emphasizing that additional studies are needed to further explore how this disease manifests in these underrepresented populations (Owen et al., 2015). With this paper, as previously stated, we call for a more cautious and inclusive approach for categorizing the pathogenicity of a genetic variant. This can only be achieved by not only sequencing non-white groups, but also phenotyping them to strive to provide more equitable healthcare access to these historically underserved groups to develop an accurate understanding of the differences in phenotypic presentation across populations.

## 5 Perspectives and conclusion

In our initial effort to better characterize the prevalence of cystinosis by assessing disease-causing variants of the *CTNS* gene in the general population, we identified a variant (c.124G>A) with a discrepancy between its pathogenic classification in HGMD by the ACMG allele frequency guideline. Notably, the variant of interest only showed this discrepancy of high allele frequency specifically in the African ancestral group while remaining low in all other populations. Given that the c.124G>A variant causes atypical cystinosis, which may not present with as severe of a phenotype as the infantile type and instead show variable expressivity, accurate diagnosis would require detailed phenotyping and long-term follow-up. This makes disparities in access to care a potential confounding variable when classifying the pathogenicity of a disease variant, as people from populations that have decreased access to long-term detailed healthcare follow-up may be more difficult to appropriately identify and diagnose.

We propose that access to healthcare is a possible confounding factor that should be considered in the categorization of pathogenicity. This is especially true for alleles that have incomplete penetrance and variable expressivity, which may manifest differently across individuals in various populations. In order to provide more equitable care across all populations, we propose a more inclusive approach—increased efforts in diagnosis in diverse populations, especially among those that may have decreased access to healthcare. Following a more inclusive approach to better understand the natural history and long-term progression of the disease, we can be more confident that subsequent phenotypic studies, such as PheWAS, can appropriately classify these variants.

## Data Availability

The original contributions presented in the study are included in the article/Supplementary Material, further inquiries can be directed to the corresponding author.

## References

[B1] 1000 Genomes Project Consortium, Abecasis G. R. Auton A. Brooks L. D. DePristo M. A. Durbin R. M. (2012). An integrated map of genetic variation from 1,092 human genomes. Nature 491 (7422), 56–65. 10.1038/nature11632 23128226 PMC3498066

[B2] 1000 Genomes Project Consortium, Auton A. Brooks L. D. Durbin R. M. Garrison E. P. Kang H. M. (2015). A global reference for human genetic variation. Nature 526 (7571), 68–74. 10.1038/nature15393 26432245 PMC4750478

[B3] Ariceta G. Giordano V. Santos F. (2019). Effects of long-term cysteamine treatment in patients with cystinosis. Pediatr. Nephrol. 34 (4), 571–578. 10.1007/s00467-017-3856-4 29260317 PMC6394685

[B4] Attard M. Jean G. Forestier L. Cherqui S. van't Hoff W. Broyer M. (1999). Severity of phenotype in cystinosis varies with mutations in the CTNS gene: predicted effect on the model of cystinosin. Hum. Mol. Genet. 8 (13), 2507–2514. 10.1093/hmg/8.13.2507 10556299

[B5] Bainbridge M. N. (2020). Determining the incidence of rare diseases. Hum. Genet. 139 (5), 569–574. 10.1007/s00439-020-02135-5 32056000 PMC7176520

[B6] Buniello A. MacArthur J. A. L. Cerezo M. Harris L. W. Hayhurst J. Malangone C. (2019). The NHGRI-EBI GWAS Catalog of published genome-wide association studies, targeted arrays and summary statistics 2019. Nucleic Acids Res. 47 (D1), D1005–D1012. 10.1093/nar/gky1120 30445434 PMC6323933

[B7] Chandra M. Stokes M. B. Kaskel F. (2010). Multinucleated podocytes: a diagnostic clue to cystinosis. Kidney Int. 78 (10), 1052. 10.1038/ki.2010.341 21030980

[B8] Chen K. Wu W. Mathew D. Zhang Y. Browne S. K. Rosen L. B. (2014). Autoimmunity due to RAG deficiency and estimated disease incidence in RAG1/2 mutations. J. Allergy Clin. Immunol. 133 (3), 880–882. 10.1016/j.jaci.2013.11.038 24472623 PMC4107635

[B9] David D. Princiero Berlingerio S. Elmonem M. A. Oliveira Arcolino F. Soliman N. van den Heuvel B. (2019). Molecular basis of cystinosis: geographic distribution, functional consequences of mutations in the CTNS gene, and potential for repair. Nephron 141 (2), 133–146. 10.1159/000495270 30554218

[B10] Doyle M. Werner-Lin A. (2015). That eagle covering me: transitioning and connected autonomy for emerging adults with cystinosis. Pediatr. Nephrol. 30 (2), 281–291. 10.1007/s00467-014-2921-5 25159720 PMC4282721

[B11] Ebbesen F. Mygind K. I. Holck F. (1976). Infantile nephropatic cystinosis in Denmark. Dan. Med. Bull. 23 (5), 216–222. 975942

[B12] Elmonem M. A. Mahmoud I. G. Mehaney D. A. Sharaf S. A. Hassan S. A. Orabi A. (2016b). Lysosomal storage disorders in Egyptian children. Indian J. Pediatr. 83 (8), 805–813. 10.1007/s12098-015-2014-x 26830282

[B13] Elmonem M. A. Veys K. R. Soliman N. A. van Dyck M. van den Heuvel L. P. Levtchenko E. (2016a). Cystinosis: a review. Orphanet J. Rare Dis. 11, 47. 10.1186/s13023-016-0426-y 27102039 PMC4841061

[B14] Fatumo S. Chikowore T. Choudhury A. Ayub M. Martin A. R. Kuchenbaecker K. (2022). A roadmap to increase diversity in genomic studies. Nat. Med. 28 (2), 243–250. 10.1038/s41591-021-01672-4 35145307 PMC7614889

[B15] Gahl W. A. (1986). Cystinosis coming of age. Adv. Pediatr. 33, 95–126. 10.1016/s0065-3101(24)00302-5 3541536

[B16] Gahl W. A. Kuehl E. M. Iwata F. Lindblad A. Kaiser-Kupfer M. I. (2000). Corneal crystals in nephropathic cystinosis: natural history and treatment with cysteamine eyedrops. Mol. Genet. Metab. 71 (1–2), 100–120. 10.1006/mgme.2000.3062 11001803

[B17] Gahl W. A. Reed G. F. Thoene J. G. Schulman J. D. Rizzo W. B. Jonas A. J. (1987). Cysteamine therapy for children with nephropathic cystinosis. N. Engl. J. Med. 316 (16), 971–977. 10.1056/NEJM198704163161602 3550461

[B18] Gahl W. A. Thoene J. G. Schneider J. A. (2002). Cystinosis. N. Engl. J. Med. 347 (2), 111–121. 10.1056/NEJMra020552 12110740

[B19] Hardy G. H. (1908). Mendelian proportions in a mixed population. Science 28 (706), 49–50. 10.1126/science.28.706.49 17779291

[B20] Harrison S. M. Biesecker L. G. Rehm H. L. (2019). Overview of specifications to the ACMG/AMP variant interpretation guidelines. Curr. Protoc. Hum. Genet. 103 (1), e93. 10.1002/cphg.93 31479589 PMC6885382

[B21] Hohenfellner K. Elenberg E. Ariceta G. Nesterova G. Soliman N. A. Topaloglu R. (2022). Newborn screening: review of its impact for cystinosis. Cells 11 (7), 1109. 10.3390/cells11071109 35406673 PMC8997957

[B22] Hopp K. Cogal A. G. Bergstralh E. J. Seide B. M. Olson J. B. Meek A. M. (2015). Phenotype-genotype correlations and estimated carrier frequencies of primary hyperoxaluria. J. Am. Soc. Nephrol. 26 (10), 2559–2570. 10.1681/ASN.2014070698 25644115 PMC4587693

[B23] Hult M. Darin N. von Döbeln U. Månsson J.-E. (2014). Epidemiology of lysosomal storage diseases in Sweden. Acta Paediatr. 103 (12), 1258–1263. 10.1111/apa.12807 25274184

[B24] Ivanova E. De Leo M. G. De Matteis M. A. Levtchenko E. (2014). Cystinosis: clinical presentation, pathogenesis and treatment. Pediatr. Endocrinol. Rev. 12 (Suppl. 1), 176–184. 25345100

[B25] Kalatzis V. Nevo N. Cherqui S. Gasnier B. Antignac C. (2004). Molecular pathogenesis of cystinosis: effect of CTNS mutations on the transport activity and subcellular localization of cystinosin. Hum. Mol. Genet. 13 (13), 1361–1371. 10.1093/hmg/ddh152 15128704

[B26] Karczewski K. J. Francioli L. C. Tiao G. Cummings B. B. Alföldi J. Wang Q. (2020). The mutational constraint spectrum quantified from variation in 141,456 humans. Nature 581 (7809), 434–443. 10.1038/s41586-020-2308-7 32461654 PMC7334197

[B27] McDowell G. A. (1995). Linkage of the gene for cystinosis to markers on the short arm of chromosome 17. The Cystinosis Collaborative Research Group. Nat. Genet. 10 (2), 246–248. 10.1038/ng0695-246 7663525

[B28] Nesterova G. Gahl W. A. (2013). Cystinosis: the evolution of a treatable disease. Pediatr. Nephrol. 28 (1), 51–59. 10.1007/s00467-012-2242-5 22903658 PMC3505515

[B29] Nießl C. Boulesteix A. L. Oh J. Palm K. Schlingmann P. Wygoda S. (2022). Relationship between age at initiation of cysteamine treatment, adherence with therapy, and glomerular kidney function in infantile nephropathic cystinosis. Mol. Genet. Metab. 136 (4), 268–273. 10.1016/j.ymgme.2022.06.010 35835062 PMC9395137

[B30] Owen E. P. Nandhlal J. Leisegang F. Van der Watt G. Nourse P. Gajjar P. (2015). Common mutation causes cystinosis in the majority of black South African patients. Pediatr. Nephrol. 30 (4), 595–601. 10.1007/s00467-014-2980-7 25326109

[B31] Regnier M. Flammier S. Boutaba M. Ndongo A. A. Servais A. Schaefer F. (2023). Worldwide disparities in access to treatment and investigations for nephropathic cystinosis: a 2023 perspective. Pediatr. Nephrol. 39, 1113–1123. 10.1007/s00467-023-06179-3 37978055 PMC10899370

[B32] Saulsberry K. Terry S. F. (2013). The need to build trust: a perspective on disparities in genetic testing. Genet. Test. Mol. Biomarkers 17 (9), 647–648. 10.1089/gtmb.2013.1548 24000888 PMC3761437

[B33] Schrodi S. J. DeBarber A. He M. Ye Z. Peissig P. Van Wormer J. J. (2015). Prevalence estimation for monogenic autosomal recessive diseases using population-based genetic data. Hum. Genet. 134 (6), 659–669. 10.1007/s00439-015-1551-8 25893794

[B34] Sumayao R. Newsholme P. McMorrow T. (2018). The role of cystinosin in the intermediary thiol metabolism and redox homeostasis in kidney proximal tubular cells. Antioxidants (Basel) 7 (12), 179. 10.3390/antiox7120179 30513914 PMC6315507

[B35] Williams D. R. Rucker T. D. (2000). Understanding and addressing racial disparities in health care. Health Care Financ. Rev. 21 (4), 75–90. 11481746 PMC4194634

[B36] Wu C.-H. W. Badreddine J. Chang J. Huang Y. R. M. Kim F. J. Wild T. (2023). Population genetics analysis of SLC3A1 and SLC7A9 revealed the etiology of cystine stone may be more than what our current genetic knowledge can explain. Urolithiasis 51 (1), 101. 10.1007/s00240-023-01473-z 37561200

