# Supplementary material for: Perspectives from cystinosis: access to healthcare may be a confounding factor for variant classification
Source: Front Genet. 2024 Jul 24;15:1402667. doi: 10.3389/fgene.2024.1402667 (PMC11303213; doi:10.3389/fgene.2024.1402667)
Supplement: Supplementary file 1 [file DataSheet2.PDF]

Supplementary Table 1. Variants of *CTNS* in the 1KG database

Chromosome	Start Position	End Position	Variant Type	Reference Genotype | Proteotype	Variant 1 Genotype | Proteotype	Count	Variant 2 Genotype | Proteotype	Count
chr17	3539835	3539835	SNV	T	C:C	4	C:T	106
chr17	3539897	3539897	SNV	C	C:T	102	T:T	4
chr17	3540042	3540042	SNV	G	A:A	43	A:G	281
chr17	3540136	3540136	SNV	A	A:G	47	G:G	2
chr17	3540163	3540163	SNV	A	A:G	49	G:G	2
chr17	3540662	3540662	deletion	T	-:-	1092		
chr17	3540693	3540693	SNV	G	A:A	169	A:G	439
chr17	3540885	3540885	SNV	C	C:T	1		
chr17	3540917	3540917	SNV	T	C:C	171	C:T	441
chr17	3541106	3541107	deletion	TC	-:-	3	-:TC	108
chr17	3541107	3541109	deletion	CTC	-:-	4	-:CTC	102
chr17	3541111	3541113	deletion	TCA	-:-	3	-:TCA	102
chr17	3541113	3541113	SNV	A	A:G	23	G:G	3
chr17	3541140	3541140	SNV	C	C:T	2		
chr17	3541143	3541145	deletion	CTT	-:-	8	-:CTT	94
chr17	3541172	3541172	deletion	A	-:-	50	-:A	172
chr17	3541208	3541208	SNV	C	A:A	4	A:C	102
chr17	3541283	3541283	SNV	C	A:C	10		
chr17	3541350	3541350	SNV	G	A:G	30		
chr17	3541431	3541431	SNV	G	G:T	26		
chr17	3541441	3541441	insertion	-	-:A	144	A:A	16
chr17	3541504	3541504	SNV	T	G:T	6		
chr17	3541766	3541766	SNV	A	A:G	283	G:G	41
chr17	3541818	3541818	SNV	C	C:T	10		
chr17	3541940	3541940	SNV	C	C:T	23	T:T	3
chr17	3542067	3542067	SNV	G	G:T	2		
chr17	3542075	3542075	SNV	G	A:A	170	A:G	442
chr17	3542119	3542119	SNV	A	A:G	3		
chr17	3542136	3542136	SNV	A	A:C	315	C:C	43

chr17	3542162	3542162	SNV	C	C:T	24	T:T	1
chr17	3542166	3542166	SNV	C	C:T	17		
chr17	3542208	3542208	SNV	G	A:G	5		
chr17	3542243	3542243	SNV	G	A:G	3		
chr17	3542283	3542283	SNV	G	C:C	48	C:G	131
chr17	3542455	3542455	SNV	T	C:C	170	C:T	441
chr17	3542479	3542479	SNV	G	A:A	170	A:G	440
chr17	3542485	3542485	SNV	C	C:T	207	T:T	65
chr17	3542524	3542524	SNV	C	C:G	442	G:G	170
chr17	3542593	3542593	SNV	C	C:G	442	G:G	170
chr17	3542626	3542626	insertion	-	-:T	69	T:T	2
chr17	3542709	3542709	SNV	C	A:A	2	A:C	35
chr17	3542768	3542768	SNV	G	C:C	166	C:G	437
chr17	3542819	3542819	SNV	G	A:G	6		
chr17	3542884	3542884	SNV	C	C:T	1		
chr17	3542895	3542895	SNV	G	A:G	1		
chr17	3542916	3542916	SNV	C	C:T	3		
chr17	3542991	3542991	deletion	A	-:-	51	-:A	149
chr17	3543204	3543204	SNV	C	C:T	2		
chr17	3543271	3543271	SNV	A	A:G	442	G:G	170
chr17	3543710	3543710	SNV	G	A:G	2		
chr17	3543855	3543855	SNV	C	C:G	1		
chr17	3543930	3543930	SNV	G	C:G	6		
chr17	3544017	3544017	SNV	C	C:T	358	T:T	123
chr17	3544039	3544039	deletion	T	-:-	4	-:T	66
chr17	3544054	3544054	SNV	C	C:T	31	T:T	2
chr17	3544191	3544191	SNV	C	A:A	1	A:C	33
chr17	3544209	3544209	SNV	A	A:T	3		
chr17	3544237	3544237	SNV	T	A:T	5		
chr17	3544295	3544295	SNV	A	A:G	442	G:G	169
chr17	3544313	3544313	SNV	G	A:A	49	A:G	130
chr17	3544405	3544405	SNV	G	G:T	13		

chr17	3544670	3544678	deletion	ATATATATA	-:-	161	-:ATATATATA	453
chr17	3544679	3544679	insertion	-	-:ATATATA	267	ATATATA:ATATATA	56
chr17	3544679	3544683	deletion	TTTTT	-:-	15	-:TTTTT	186
chr17	3544729	3544729	SNV	A	A:G	443	G:G	172
chr17	3544749	3544749	SNV	G	A:G	5		
chr17	3544779	3544779	SNV	G	A:A	171	A:G	441
chr17	3544835	3544835	SNV	G	A:A	50	A:G	131
chr17	3545030	3545030	SNV	C	C:G	5		
chr17	3545112	3545112	SNV	G	A:A	218	A:G	455
chr17	3545127	3545127	SNV	G	A:A	171	A:G	440
chr17	3545233	3545233	SNV	G	A:A	1	A:G	11
chr17	3545302	3545302	SNV	T	C:C	218	C:T	455
chr17	3545315	3545315	SNV	G	A:A	205	A:G	460
chr17	3545323	3545323	SNV	A	A:G	458	G:G	215
chr17	3545385	3545386	deletion	GT	-:-	9	-:GT	90
chr17	3545416	3545416	SNV	T	C:C	1061	C:T	31
chr17	3545515	3545515	SNV	C	C:G	1		
chr17	3545529	3545529	SNV	A	A:G	458	G:G	216
chr17	3545559	3545559	SNV	T	C:C	219	C:T	454
chr17	3545810	3545810	SNV	C	C:T	343	T:T	121
chr17	3545811	3545811	SNV	G	A:G	25		
chr17	3545851	3545851	SNV	A	A:G	403	G:G	540
chr17	3545873	3545873	SNV	T	C:T	16		
chr17	3546030	3546030	SNV	C	C:T	1		
chr17	3546093	3546093	SNV	G	A:G	2		
chr17	3546103	3546103	SNV	A	A:G	1		
chr17	3546130	3546130	SNV	C	A:C	1		
chr17	3546153	3546153	SNV	T	C:C	1	C:T	10
chr17	3546160	3546160	SNV	G	A:A	123	A:G	355
chr17	3546195	3546195	SNV	C	C:G	23	G:G	3
chr17	3546231	3546231	SNV	T	C:C	1033	C:T	57
chr17	3546347	3546347	SNV	G	A:A	216	A:G	456

chr17	3546410	3546410	SNV	T	C:C	1	C:T	58
chr17	3546424	3546424	SNV	T	C:T	6		
chr17	3546476	3546476	SNV	C	C:T	82	T:T	2
chr17	3546557	3546557	SNV	G	A:G	1		
chr17	3546639	3546639	SNV	T	C:C	171	C:T	440
chr17	3546665	3546665	SNV	G	A:G	28		
chr17	3546667	3546667	SNV	T	C:T	1		
chr17	3546887	3546890	deletion	CTCA	-:-	144	-:CTCA	379
chr17	3546921	3546921	SNV	C	C:T	1		
chr17	3547105	3547105	SNV	A	A:C	467	C:C	229
chr17	3547186	3547186	SNV	T	C:C	216	C:T	466
chr17	3547187	3547187	SNV	G	A:G	1		
chr17	3547318	3547318	SNV	T	G:T	3		
chr17	3547451	3547451	SNV	T	C:T	1		
chr17	3547580	3547580	SNV	G	A:G	4		
chr17	3547583	3547583	SNV	C	C:T	7		
chr17	3547781	3547781	insertion	-	-:T	132	T:T	27
chr17	3547832	3547832	SNV	C	C:T	1		
chr17	3547844	3547844	SNV	C	C:T	7		
chr17	3548199	3548199	SNV	A	A:G	130	G:G	49
chr17	3548333	3548333	SNV	T	C:T	3		
chr17	3548447	3548447	SNV	A	A:G	468	G:G	229
chr17	3548462	3548462	SNV	C	A:C	1		
chr17	3548617	3548617	SNV	G	A:G	1		
chr17	3548628	3548628	SNV	T	G:T	1		
chr17	3548647	3548647	SNV	C	C:T	467	T:T	230
chr17	3548689	3548689	SNV	G	A:A	230	A:G	467
chr17	3548720	3548720	SNV	G	A:G	2		
chr17	3548784	3548784	SNV	C	C:T	376	T:T	144
chr17	3548806	3548806	SNV	C	C:T	1		
chr17	3548829	3548829	SNV	G	A:A	124	A:G	344
chr17	3548981	3548981	SNV	T	C:T	18		

chr17	3549227	3549227	SNV	G	C:C	2	C:G	20
chr17	3549239	3549239	SNV	T	A:T	14		
chr17	3549260	3549260	deletion	A	-:-	5	-:A	115
chr17	3549267	3549267	deletion	C	-:-	55	-:C	257
chr17	3549268	3549268	SNV	C	A:A	173	A:C	445
chr17	3549270	3549270	deletion	G	-:-	183	-:G	453
chr17	3549300	3549300	SNV	G	A:A	125	A:G	358
chr17	3549405	3549405	SNV	G	A:G	1		
chr17	3549470	3549470	SNV	T	C:C	417	C:T	454
chr17	3549543	3549543	SNV	T	A:T	3		
chr17	3549589	3549589	insertion	-	-:C	87	C:C	4
chr17	3549615	3549615	SNV	C	C:G	35	G:G	2
chr17	3549797	3549797	SNV	G	A:G	1		
chr17	3549826	3549826	SNV	C	C:G	31	G:G	1
chr17	3549871	3549871	SNV	A	A:G	81	G:G	1
chr17	3550082	3550082	insertion	-	-:G	122	G:G	870
chr17	3550133	3550133	SNV	G	A:G	1		
chr17	3550163	3550163	SNV	T	A:A	2	A:T	12
chr17	3550252	3550252	SNV	C	C:G	1		
chr17	3550284	3550284	SNV	C	C:G	101	G:G	5
chr17	3550396	3550396	SNV	C	C:T	101	T:T	5
chr17	3550408	3550408	SNV	A	A:G	134	G:G	899
chr17	3550409	3550409	SNV	C	C:T	12		
chr17	3550514	3550514	SNV	C	C:T	73	T:T	2
chr17	3550520	3550520	SNV	G	A:G	1		
chr17	3550784	3550784	SNV	C|N	C:T | N:N	8	T:T | N:N	1
chr17	3550792	3550792	SNV	C|S	C:T | S:L	2		
chr17	3550799	3550799	SNV	C|N	C:T | N:N	3		
chr17	3550800	3550800	SNV	G|V	A:G | I:V	35		
chr17	3550842	3550842	SNV	C	C:T	1		
chr17	3550850	3550850	SNV	C	C:T	1		
chr17	3551123	3551123	SNV	C	C:T	5		

chr17	3551178	3551178	SNV	A	A:G	154	G:G	835
chr17	3551199	3551199	deletion	T	-:-	2	-:T	44
chr17	3551310	3551310	SNV	C	C:T	284	T:T	41
chr17	3551327	3551327	SNV	T	C:C	863	C:T	166
chr17	3551345	3551345	SNV	T	G:G	124	G:T	358
chr17	3551586	3551586	SNV	C	C:T	3		
chr17	3551616	3551616	SNV	T	C:C	1092		
chr17	3551799	3551799	SNV	C	C:T	5		
chr17	3551981	3551981	SNV	C	C:T	2		
chr17	3551994	3551994	SNV	T	G:T	1		
chr17	3551998	3551998	SNV	C	C:T	2		
chr17	3552038	3552038	SNV	G	A:A	144	A:G	375
chr17	3552261	3552261	SNV	G	A:G	1		
chr17	3552372	3552372	SNV	G	C:C	124	C:G	357
chr17	3552388	3552388	SNV	G	A:G	2		
chr17	3552389	3552389	SNV	C	A:C	2		
chr17	3552466	3552466	SNV	G	A:G	3		
chr17	3552532	3552532	SNV	A	A:G	52	G:G	8
chr17	3552678	3552678	SNV	G	A:G	3		
chr17	3552727	3552727	SNV	C	C:T	281	T:T	40
chr17	3552815	3552815	SNV	A	A:T	1		
chr17	3552890	3552890	SNV	A	A:G	16		
chr17	3553012	3553012	SNV	C	A:C	16		
chr17	3553056	3553056	insertion	-	-:C	427	C:C	248
chr17	3553058	3553058	insertion	-	-:T	407	T:T	278
chr17	3553148	3553148	SNV	C	C:T	1		
chr17	3553270	3553270	SNV	A	A:G	16		
chr17	3553292	3553292	SNV	A	A:C	42	C:C	1
chr17	3553358	3553358	SNV	A	A:G	1		
chr17	3553426	3553426	SNV	A	A:T	1		
chr17	3553430	3553430	SNV	A	A:G	16		
chr17	3553451	3553451	SNV	A	A:G	3		

chr17	3553474	3553474	SNV	G	A:G	16		
chr17	3553477	3553477	SNV	G	A:G	1		
chr17	3553489	3553489	SNV	G	A:G	1		
chr17	3553526	3553526	SNV	A	A:G	166	G:G	863
chr17	3553582	3553582	SNV	G	A:G	19		
chr17	3553655	3553655	SNV	A	A:G	85	G:G	5
chr17	3553665	3553665	SNV	A	A:G	37	G:G	1
chr17	3553743	3553743	SNV	T	C:C	2	C:T	10
chr17	3553766	3553766	SNV	A	A:C	12		
chr17	3554009	3554009	SNV	G	A:A	1	A:G	37
chr17	3554059	3554059	SNV	G	A:A	375	A:G	462
chr17	3554136	3554136	SNV	G	A:G	2		
chr17	3554182	3554182	SNV	T	C:T	16		
chr17	3554295	3554295	SNV	C	C:T	15		
chr17	3554552	3554552	SNV	C	C:G	37	G:G	1
chr17	3554631	3554631	SNV	C	C:T	14	T:T	1
chr17	3554669	3554669	SNV	G	A:A	831	A:G	146
chr17	3554721	3554721	SNV	A	A:G	6		
chr17	3554812	3554812	SNV	C	C:T	10	T:T	1
chr17	3554817	3554817	SNV	G	A:A	1	A:G	44
chr17	3554941	3554941	SNV	A	A:G	2		
chr17	3554993	3554993	SNV	C	C:T	8		
chr17	3554994	3554994	SNV	C	C:G	160	G:G	863
chr17	3554997	3554997	deletion	G	-:-	2	-:G	76
chr17	3555153	3555153	SNV	A	A:G	2		
chr17	3555277	3555277	deletion	G	-:-	843	-:G	182
chr17	3555288	3555288	SNV	A	A:T	16		
chr17	3555289	3555289	SNV	A	A:C	16		
chr17	3555390	3555390	SNV	A	A:C	16		
chr17	3555424	3555424	SNV	G	A:A	40	A:G	281
chr17	3555439	3555439	SNV	G	A:G	13		
chr17	3555492	3555492	SNV	A	A:C	6		

chr17	3555624	3555624	SNV	G	C:G	4		
chr17	3555652	3555652	SNV	G	A:A	2	A:G	80
chr17	3555747	3555747	SNV	G	A:G	3		
chr17	3555754	3555754	SNV	C	C:T	1		
chr17	3555817	3555817	SNV	C	C:T	172	T:T	843
chr17	3555867	3555867	SNV	C	C:T	4		
chr17	3555894	3555894	SNV	A	A:T	3		
chr17	3555915	3555915	SNV	C	C:T	3		
chr17	3555986	3555986	SNV	T	C:C	863	C:T	166
chr17	3556120	3556120	SNV	T	C:T	16		
chr17	3556140	3556140	SNV	T	C:T	16		
chr17	3556150	3556150	insertion	-	-:C	148	C:C	9
chr17	3556292	3556292	SNV	G	A:G	3		
chr17	3556324	3556324	SNV	C	C:T	3		
chr17	3556432	3556432	SNV	A	G:G	1092		
chr17	3556456	3556456	SNV	A	A:G	27	G:G	2
chr17	3556463	3556463	SNV	T	C:T	16		
chr17	3556471	3556471	SNV	C	C:T	14		
chr17	3556494	3556494	SNV	T	C:T	1		
chr17	3556498	3556498	SNV	A	A:G	1		
chr17	3556501	3556501	SNV	A	A:T	1		
chr17	3556504	3556504	SNV	G	A:G	1		
chr17	3556563	3556563	SNV	T	C:C	861	C:T	170
chr17	3556594	3556594	SNV	C	C:T	313	T:T	108
chr17	3556600	3556600	SNV	A	A:G	16		
chr17	3556623	3556623	SNV	C	C:G	12		
chr17	3556782	3556782	SNV	A	A:G	3		
chr17	3556832	3556832	SNV	G	A:G	1		
chr17	3556897	3556897	SNV	C	C:T	358	T:T	142
chr17	3556920	3556920	SNV	A	A:G	16		
chr17	3556983	3556983	SNV	A	A:G	2		
chr17	3557042	3557042	SNV	C	C:T	1		

chr17	3557055	3557055	SNV	A	A:T	7		
chr17	3557076	3557076	SNV	G	A:G	1		
chr17	3557131	3557131	SNV	T	C:T	19		
chr17	3557175	3557175	insertion	-	-:A	95	A:A	978
chr17	3557181	3557181	deletion	T	-:-	861	-:T	168
chr17	3557219	3557219	SNV	T	C:T	7		
chr17	3557381	3557381	SNV	A	A:T	1		
chr17	3557382	3557382	SNV	A	A:T	164	T:T	867
chr17	3557495	3557495	SNV	A	A:G	387	G:G	145
chr17	3557499	3557499	SNV	C	C:T	345	T:T	123
chr17	3557537	3557537	SNV	C	C:T	158	T:T	859
chr17	3557594	3557594	SNV	A	A:G	158	G:G	859
chr17	3557601	3557601	SNV	C	C:T	13	T:T	1
chr17	3557647	3557647	SNV	G	G:T	1		
chr17	3557837	3557837	SNV	G	A:G	4		
chr17	3557846	3557846	SNV	T	G:G	1	G:T	34
chr17	3557890	3557890	SNV	G	A:G	1		
chr17	3558024	3558024	SNV	C	C:T	1		
chr17	3558098	3558098	SNV	G	A:G	16		
chr17	3558185	3558185	SNV	C	C:T	11	T:T	1
chr17	3558212	3558212	SNV	C	A:C	3		
chr17	3558266	3558266	SNV	G	A:G	2		
chr17	3558417	3558417	SNV	C	C:G	166	G:G	
chr17	3558426	3558426	SNV	C	A:C	1		
chr17	3558443	3558443	SNV	C	C:T	4		
chr17	3558467	3558467	SNV	T	C:T	2		
chr17	3558486	3558486	SNV	C	C:T	1		
chr17	3558549	3558549	SNV	G|A	A:G | T:A	1		
chr17	3558553	3558553	SNV	T|I	G:T | S:I	1		
chr17	3558688	3558688	SNV	T	C:T	1		
chr17	3558695	3558695	SNV	A	A:G	386	G:G	145
chr17	3558698	3558698	SNV	G	A:A	378	A:G	472

chr17	3558700	3558700	SNV	G	A:A	46	A:G	301
chr17	3558760	3558760	SNV	A	A:G	3		
chr17	3558963	3558963	SNV	C	A:A	4	A:C	85
chr17	3559273	3559273	SNV	G	G:T	5	T:T	1
chr17	3559303	3559303	SNV	T	G:G	2	G:T	57
chr17	3559430	3559430	SNV	G	A:A	2	A:G	89
chr17	3559451	3559451	SNV	T	C:C	12	C:T	199
chr17	3559481	3559481	SNV	A	A:C	235	C:C	18
chr17	3559502	3559502	SNV	T	C:C	100	C:T	353
chr17	3559506	3559506	SNV	T	C:C	101	C:T	350
chr17	3559511	3559511	SNV	A	A:C	403	C:C	88
chr17	3559517	3559517	SNV	C	A:A	137	A:C	347
chr17	3559651	3559651	SNV	C	C:T	235	T:T	42
chr17	3559676	3559677	deletion	CT	-:-	20	-:CT	169
chr17	3559687	3559687	SNV	C	C:T	246	T:T	22
chr17	3559722	3559722	SNV	T	C:T	5		
chr17	3559742	3559742	SNV	T	C:T	1		
chr17	3559774	3559774	SNV	C	A:C	7		
chr17	3559781	3559781	SNV	T|S	C:C | S:S		C:T | S:S	89
chr17	3559792	3559792	SNV	T|L	C:T | P:L	1		
chr17	3559806	3559806	SNV	G|V	A:G | M:V	1		
chr17	3559823	3559823	SNV	G|T	A:A | T:T	39	A:G | T:T	281
chr17	3559884	3559884	SNV	C	C:T	1		
chr17	3559901	3559901	SNV	C	A:C	1		
chr17	3560009	3560009	SNV	G|V	A:G | M:V	1		
chr17	3560020	3560020	SNV	C|N	C:T | N:N	2		
chr17	3560110	3560110	SNV	C	A:C	2		
chr17	3560363	3560363	SNV	G	A:A	47	A:G	306
chr17	3560371	3560377	deletion	AGGGAGG	-:-	11	-:AGGGAGG	115
chr17	3560450	3560450	SNV	G	A:G	1		
chr17	3560541	3560542	deletion	GA	-:-	5	-:GA	30
chr17	3560561	3560561	SNV	G	G:T	3		

chr17	3560598	3560598	SNV	G	A:A	50	A:G	129
chr17	3560683	3560683	SNV	G	A:A	13	A:G	67
chr17	3560752	3560752	SNV	T	C:C	1030	C:T	61
chr17	3560987	3560987	SNV	G	A:G	2		
chr17	3561200	3561200	SNV	C	C:T	1		
chr17	3561201	3561201	SNV	G	A:A	3	A:G	54
chr17	3561233	3561233	SNV	C	A:A	3	A:C	54
chr17	3561249	3561249	SNV	C	C:T	2		
chr17	3561302	3561302	SNV	G|G	A:G | S:G	2		
chr17	3561312	3561312	SNV	G|R	A:G | H:R	2		
chr17	3561396	3561396	SNV	C|T	C:T | T:I	210	T:T | I:I	812
chr17	3561451	3561451	SNV	G|L	A:G | L:L	1		
chr17	3561671	3561671	SNV	G	A:G	3		
chr17	3561697	3561697	SNV	G	A:A	1	A:G	59
chr17	3561711	3561711	SNV	C	C:G	404	G:G	434
chr17	3561712	3561712	SNV	A	A:G	402	G:G	436
chr17	3561725	3561725	SNV	G	A:G	3		
chr17	3561728	3561728	SNV	C	C:G	176	G:G	61
chr17	3561744	3561744	SNV	C	C:T	16		
chr17	3561750	3561750	SNV	T	C:C	56	C:T	177
chr17	3561765	3561765	SNV	G	A:G	3		
chr17	3561817	3561817	SNV	G	A:G	21		
chr17	3561842	3561842	SNV	C	C:T	448	T:T	145
chr17	3561912	3561912	SNV	A	A:T	6		
chr17	3561956	3561956	SNV	C	C:T	348	T:T	52
chr17	3561961	3561961	SNV	G	A:A	1	A:G	5
chr17	3562040	3562040	SNV	C	C:G	376	G:G	140
chr17	3562182	3562182	SNV	G	A:A	975	A:G	104
chr17	3562196	3562196	SNV	G	A:A	52	A:G	335
chr17	3562200	3562200	SNV	T	C:C	50	C:T	339
chr17	3562202	3562202	SNV	T	C:C	50	C:T	334
chr17	3562405	3562405	SNV	G	A:G	11		

chr17	3562485	3562485	insertion	-	-:C	481	C:C	260
chr17	3562486	3562486	insertion	-	-:G	412	G:G	425
chr17	3562488	3562488	insertion	-	-:C	496	C:C	228
chr17	3562573	3562573	SNV	C	C:T	3		
chr17	3562617	3562617	SNV	C	C:T	2		
chr17	3562624	3562624	SNV	G	G:T	57	T:T	3
chr17	3562818	3562819	deletion	AT	-:-	3	-:AT	73
chr17	3562916	3562916	SNV	G	A:G	7		
chr17	3563009	3563009	SNV	C	C:T	1		
chr17	3563091	3563091	SNV	G	A:G	2		
chr17	3563124	3563124	SNV	C	C:T	10		
chr17	3563125	3563125	SNV	G	A:G	2		
chr17	3563196	3563196	SNV	T|I	C:T | I:I	1		
chr17	3563220	3563220	SNV	C|T	C:T | T:T	1		
chr17	3563284	3563284	SNV	G	A:A	1	A:G	49
chr17	3563314	3563314	SNV	C	C:T	131	T:T	48
chr17	3563339	3563339	SNV	C	C:T	373	T:T	138
chr17	3563416	3563416	SNV	G	A:G	3		
chr17	3563550	3563550	SNV	G|G	A:G | R:G	1		
chr17	3563588	3563588	SNV	C|I	C:T | I:I	1		
chr17	3563743	3563743	SNV	G	A:G	1		
chr17	3563923	3563923	SNV	G|A	G:T | A:A	1		
chr17	3563925	3563925	SNV	G|R	A:G | H:R	2		
chr17	3563963	3563963	SNV	C|P	C:G | P:A	329	G:G | A:A	104
chr17	3563976	3563976	SNV	C|P	C:T | P:L	2		
chr17	3563992	3563992	SNV	A|Q	A:G | Q:Q	3		
chr17	3564037	3564037	SNV	C	C:T	1		
chr17	3564068	3564068	SNV	G	A:A	52	A:G	347
chr17	3564073	3564073	SNV	A	A:G	348	G:G	52
chr17	3564084	3564084	SNV	T	C:C	52	C:T	347
chr17	3564132	3564132	SNV	G	A:G	1		
chr17	3564190	3564190	SNV	G	A:A	12	A:G	83

chr17	3564218	3564218	SNV	T	C:C	52	C:T	347
chr17	3564235	3564235	SNV	A	A:G	347	G:G	52
chr17	3564290	3564290	SNV	T	C:C	52	C:T	346
chr17	3564294	3564294	SNV	T	C:C	52	C:T	346
chr17	3564351	3564351	SNV	G	A:G	1		
chr17	3564378	3564378	SNV	C	C:T	346	T:T	51
chr17	3564385	3564385	SNV	C	C:T	1		
chr17	3564400	3564401	deletion	TT	-:-	83	-:TT	268
chr17	3564400	3564400	SNV	T	A:A	125	A:T	359
chr17	3564401	3564401	SNV	T	G:G	1086	G:T	6
chr17	3564498	3564498	SNV	A	A:C	6		
chr17	3564510	3564510	SNV	C	C:T	8		
chr17	3564514	3564514	SNV	G	A:A	3	A:G	29
chr17	3564605	3564605	SNV	C	C:T	4		
chr17	3564660	3564660	SNV	C	C:T	1		
chr17	3564687	3564687	SNV	C	C:T	288	T:T	43
chr17	3564688	3564688	SNV	A	A:G	288	G:G	42
chr17	3564693	3564693	insertion	-	-:GTC	290	GTC:GTC	45
chr17	3564708	3564708	insertion	-	-:G	132	G:G	7
chr17	3564711	3564711	insertion	-	-:G	211	G:G	24
chr17	3564716	3564716	insertion	-	-:G	224	G:G	25
chr17	3564753	3564753	SNV	T	C:T	1		
chr17	3564779	3564779	SNV	C	C:T	329	T:T	52
chr17	3564780	3564780	SNV	G	A:A	1	A:G	32
chr17	3564791	3564791	SNV	G	A:G	11		
chr17	3564815	3564815	SNV	A	A:G	327	G:G	
chr17	3564823	3564823	SNV	G	A:G	42		
chr17	3564828	3564828	SNV	C	C:T	42	T:T	1
chr17	3564837	3564838	deletion	CT	-:-	52	-:CT	329
chr17	3564869	3564869	SNV	T	C:C	2	C:T	12
chr17	3564945	3564945	SNV	C	C:T	1		
chr17	3564946	3564946	SNV	G	A:G	3		

chr17	3564951	3564951	SNV	G	A:G	3		
chr17	3565103	3565103	SNV	A	A:G	398	G:G	585
chr17	3565144	3565144	SNV	T	C:T	478		
chr17	3565241	3565241	SNV	C	C:G	32	G:G	1
chr17	3565258	3565258	SNV	C	C:T	56	T:T	1
chr17	3565281	3565281	SNV	C	C:G	2		
chr17	3565296	3565296	SNV	C	C:G	56	G:G	1
chr17	3565328	3565328	SNV	A	A:G	72	G:G	1
chr17	3565331	3565331	SNV	G	A:G	1		
chr17	3565332	3565332	SNV	C	C:T	15		
chr17	3565408	3565408	SNV	C	C:T	1		
chr17	3565474	3565474	SNV	G	A:G	10		
chr17	3565529	3565529	SNV	C	C:T	54	T:T	1
chr17	3565622	3565622	SNV	T	C:T	6		
chr17	3565675	3565675	SNV	C	C:T	10	T:T	1
chr17	3565699	3565699	SNV	G	A:G	3		
chr17	3565706	3565706	SNV	G	G:T	5		
chr17	3565745	3565745	SNV	C	C:T	4		
chr17	3565778	3565778	SNV	G	A:A	1	A:G	54
chr17	3565782	3565782	SNV	G	G:T	6		
chr17	3565790	3565790	SNV	G	A:A	1	A:G	24
chr17	3565795	3565795	SNV	G	A:G	6		
chr17	3565872	3565873	deletion	GG	-:-	1	-:GG	23
chr17	3565964	3565964	SNV	C	C:T	3		
chr17	3565969	3565969	SNV	T	C:C	554	C:T	370
chr17	3566021	3566021	SNV	T	A:T	1		
chr17	3566045	3566045	insertion	-	-:C	334	C:C	690
chr17	3566066	3566066	SNV	T	G:T	1		
chr17	3566209	3566209	SNV	G	C:C	40	C:G	271
chr17	3566213	3566213	SNV	G	A:A	40	A:G	271
chr17	3566232	3566232	SNV	C	C:T	460	T:T	164
