# Supplementary figures and images for: Perspectives from cystinosis: access to healthcare may be a confounding factor for variant classification
Source: Front Genet. 2024 Jul 24;15:1402667. doi: 10.3389/fgene.2024.1402667 (PMC11303213; doi:10.3389/fgene.2024.1402667)

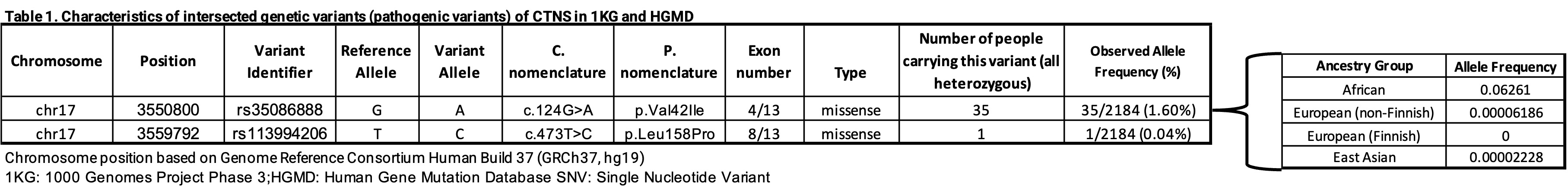

Supplement: Supplementary file 2 [file Image1.JPEG]
